# A national pilot program for chronic diseases and health inequalities in South Korea

**DOI:** 10.1186/s12889-021-11208-7

**Published:** 2021-06-15

**Authors:** Rangkyoung Ha, Dongjin Kim, Jihee Choi, Kyunghee Jung-Choi

**Affiliations:** 1 Department of Health Policy and Management, Graduate School of Public Health, Seoul National University, Seoul, 08826 Republic of Korea; 2 Korea Institute for Health and Social Affairs, Sejong, Republic of Korea; 3 Department of Occupational and Environmental Medicine, College of Medicine, Ewha Womans University School of Medicine, 25, Magokdong-ro 2-gil, Gangseo-gu, Seoul, 07804 Republic of Korea

**Keywords:** Socioeconomic position, Area deprivation, Health inequality, Continuity of prescription medication, Chronic disease management, Pilot program

## Abstract

**Background:**

To achieve the health equity, it is important to reduce socioeconomic inequalities when managing chronic diseases. In South Korea, a pilot program for chronic diseases was implemented at the national level. This study aimed to examine its effect on socioeconomic inequalities in chronic disease management at the individual and regional levels.

**Methods:**

Korean National Health Insurance data from September 2016 to October 2017 were used. Study subjects in the national pilot program for chronic diseases included 31,765 participants and 5,741,922 non-participants. The dependent variable was continuity of prescription medication. Socioeconomic position indicators were health insurance contribution level and the area deprivation index. Covariates were gender, age, and the Charlson Comorbidity Index (CCI). A multilevel logistic regression model was used to address the effects at both the individual and regional levels. This is a cross-sectional study.

**Results:**

Unlike the group of non-participants, the participants showed no inequality in prescription medication continuity according to individual-level socioeconomic position. However, continuity of prescription medication was higher among those in less deprived areas compared to those in more deprived areas in both the participation and non-participation groups.

**Conclusions:**

This study found that the pilot program for chronic diseases at the least did not contribute to the worsening of health inequalities at the individual level in South Korea. However, there was a trend showing health inequalities based on the socioeconomic level of the area. These findings suggest that additional policy measures are needed to attain equality in the management of chronic diseases regardless of the regional socioeconomic position.

**Supplementary Information:**

The online version contains supplementary material available at 10.1186/s12889-021-11208-7.

## Background

Health ensures fair opportunities [1] and is an essential element of human development [2]. Ethical value judgments are involved in this meaning of health. Social inequalities in health are socially constituted by everyday living conditions in which a person is born, grows, lives, works and ages [3]. Thus, health inequalities are not consistent with social justice [4] and are an important issue that should be approached with fairness [5]. Although efforts have been made to resolve such health inequalities in the world since the 2000s [3, 5, 6], these inequalities have not been mitigated [7]. According to the Marmot Review [5], the overall health of the UK population has improved since 1995; however, disparities in life expectancy at birth based on area and at the individual level have worsened.

The situation in South Korea is similar. Life expectancy at birth increased from 78.24 years in 2005 to 82.36 years in 2016 [8]; however, life expectancy at birth differed depending on education and income level. In 2010, the life expectancy of people aged 30 with a minimum of a college degree was 8.1 years higher than of those who only graduated from middle school [9]. The difference in life expectancy between high- and low-income earners was 6.22 years among men and 1.74 years among women [10]. There were also regional disparities in life expectancy with metropolitan areas having relatively high life expectancies [9].

According to Korean researches, the prevalence of diabetes mellitus and hypertension also differed according to socioeconomic position [11]. A lower prevalence was found in those with higher education and income levels [12–14]. According to a previous study, obesity, an unhealthy diet and lack of physical activity were well-known risk factors for chronic diseases, such as hypertension or diabetes [15], and these conditions were more common in people with low socioeconomic positions [16]. Low socioeconomic levels may be associated with a higher prevalence of diabetes or hypertension due to limited access to information on health behavior and environmental exposures [17] or a lack of healthy foods [18]. Socioeconomic position determines the distribution of resources (e.g., power, income, and education), and exposures (e.g., living conditions, working environment, and community environment). The distribution of these resources and exposure factors and the interaction between the two formulate material circumstances, health behaviors, and psychosocial factors, which ultimately result in health inequalities [19]. People of a lower socioeconomic position were likely to experience housing instability [20]. The social determinants of health were also related to the incidence of diabetes [21, 22]. Moreover, a previous study reported poverty as a major contributor to the increased prevalence of diabetes [23]. Health inequalities do not occur accidentally but are influenced by institutional, political, and economic contexts [19].

There were also inequalities in healthcare utilization among patients with chronic diseases. Several studies have found that income and education level affect healthcare utilization [24, 25]. The Horizontal Inequity Index (HI) adjusted for health needs indicated that high-income patients used relatively more healthcare services than low-income patients [24, 26].

In South Korea, as in any Western society, diabetes and hypertension have become common diseases. The prevalence of hypertension and diabetes increased from 24.4 and 9.5% in 2007 to 29.1 and 11.3% in 2016, respectively, in people over 30 years of age [27]. Many pilot programs have attempted to effectively manage the increasing number of patients with diabetes or hypertension, including a pilot program for chronic diseases by the Ministry of Health and Welfare (2016–2018). This program aimed to reduce the incidence of diabetes and hypertension complications, enhance self-management through non-face-to-face management, and strengthen primary healthcare through clinic use. When an outpatient visited a primary medical institution, a healthcare professional would encourage him or her to participate in this program. If they agreed, they were registered as a program participant [28]. The main services included establishing monthly patient chronic disease management plans through face-to-face care and providing feedback to patients after they sent blood pressure and blood sugar level data to healthcare professionals for analysis. In addition, this program provided continuous observation through non-face-to-face management and phone consultations to encourage patients’ self-management skills if necessary. After monitoring, the patient’s health was evaluated, and the results were implemented into their care plan. Patients who participated in this pilot program were exempt from copayments [29].

Despite program implementation at the national level for chronic diseases management, there are limited studies on whether it contributed to reducing health inequalities. Previous studies have shown the effectiveness of the pilot program for chronic diseases through evaluation of patient satisfaction and self-monitoring of blood pressure and blood glucose levels [30, 31]. However, to our knowledge, there is an insufficient number of published studies targeted at identifying whether the pilot program for chronic diseases has helped mitigate health inequalities. Therefore, we aimed to confirm whether there are inequalities in participation and continuous management of chronic diseases according to individual and regional socioeconomic position. Our main objective was to identify whether there are inequalities in the prescription medication continuity among non-participants and participants of the pilot program with different socioeconomic positions at the individual and regional levels.

## Methods

### Data and study subjects

We used National Health Insurance data collected by the National Health Insurance Service (NHIS) in South Korea [32]. The National Health Insurance (NHI) is compulsory and covers about 97% of the entire Korean population [33]. The NHIS has maintained information on those insured by NHI since 2002 and has constructed the National Health Information Database (NHID) based on this data. The NHID categories include eligibility, health screening, healthcare utilization, and healthcare provider database. The eligibility database includes residency, gender, age, income-based insurance contribution, and death records. The healthcare utilization database comprises medical histories, including data on procedures, surgeries, and treatment costs as well as inpatient and outpatient prescription records. The healthcare provider database contains provider information, types of providers available, number of providers, and number of beds, as well as institutional data [34]. We analyzed NHID customized data and used the information regarding eligibility, healthcare utilization, and healthcare provider. This study follows a cross sectional design.

According to the International Classification of Diseases version 10 (ICD-10) [35], hypertension code I10–13 or diabetes mellitus code E10-E14 were selected. We included outpatients who were prescribed medication for hypertension or diabetes at least once from outpatient clinics between September of 2016 and October of 2017. The total number of outpatients with hypertension or diabetes was 6,358,623. Among them, the patients who participated in the pilot program for chronic diseases were labeled as participants, and those who did not participate were labeled as non-participants.

**Fig. 1:**
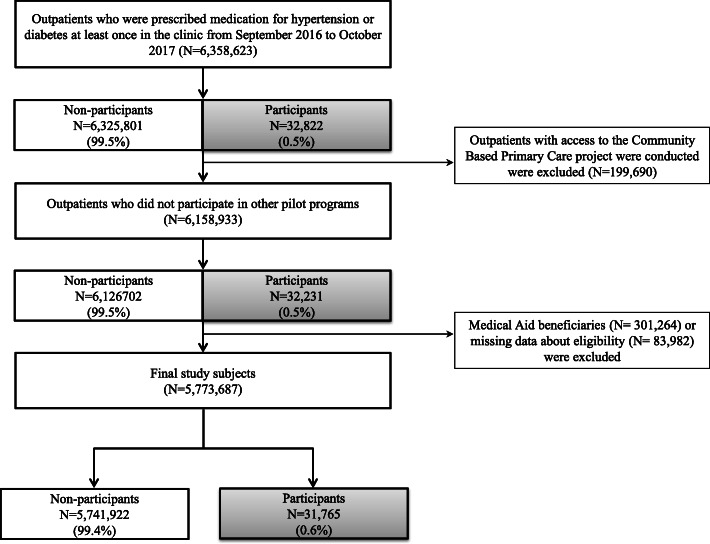
Flow chart of study subjects

To confirm the pilot program’s effectiveness, 199, 690 outpatients living in regions where another pilot program - the Community-Based Primary Care project - was carried out, were excluded because the subjects of this project were also outpatients with hypertension or diabetes. The Community-Based Primary Care project was implemented in only four regions: Jungnang-gu, Seoul; Wonju, Gangwon-do; Jeonju-si, Jeollabuk-do; and Muju-gun, Jeollabuk-do. Among these, we further excluded 301,264 medical aid beneficiaries and 83,982 individuals with missing eligibility data. Our final study subjects included 5,773,687 outpatients, comprising 31,765 participants and 5,741,922 non-participants (Fig. 1).

### Study variables

We used prescription medication continuity as the dependent variable because medication persistence is an important factor in managing hypertension and diabetes [36, 37]. Continuity of prescription medication was a dichotomous variable defined as prescription continuation when the sum of the number of days on the prescription was greater than or equal to the difference between the prescription start date and the end date of data collection (October 30, 2017).

The explanatory variables consisted of socioeconomic position: income-based insurance contribution and area deprivation. As health insurance contributions in South Korea are based on income, contribution level can be used as a proxy of income level. Health insurance contribution level is vigintile divided into 20 groups based on the number of people insured [38]. We used the sample data from the 2015 Population and Housing Census to measure area deprivation in a South Korean context [39]. The Population and Housing Census is a national basic statistics survey conducted every 5 years to gather data on the Korean population and houses. This survey is for all Korean citizens and foreign nationals residing in South Korea [40]. The area deprivation index included the assessment of low social class, poor housing conditions, low education level, no car ownership, divorce or bereavement, one-person households, female heads of household, the elderly population, and residents not living in an apartment. The area deprivation index was classified into quintiles, with Q1 being the most deprived and Q5 the least deprived.

Covariates were classified into demographic factors and clinical characteristics. Demographic factors included gender and age. Clinical characteristics consisted of the Charlson Comorbidity Index (CCI), which was originally developed to predict mortality [41], but has been widely used as a severity-adjustment method [42]. CCI assigned a weighted score to 17 conditions classified by ICD-10. Weighted scores ranged from 1 to 6, and the sum of the weights is the Charlson score [43]. The scores measured by CCI were divided into 0, 1, 2, and 3 points or more.

### Statistical analysis

Descriptive analyses were carried out to identify general characteristics. To confirm socioeconomic inequalities based on participation in the pilot program, we calculated age-standardized participation rates according to health insurance contribution levels and the area deprivation index. We calculated age-standardized continuity of prescription medication rates, and then constructed a multilevel model for the hierarchical data for both the participant and non-participant groups. A multilevel model considering both the individual and group levels can reflect within- and between-group variations [44]. We built an empty model (Model 0) that included clinics as a random intercept. Next, we included individual factors in Model 1 to investigate the extent to which continuity of prescription medication was explained by individual characteristics, such as gender, age, and CCI. Finally, we built Model 2 by adding an area deprivation variable to address the effect at the regional level.

We conducted a multilevel logistic regression analysis using the SAS GLIMMIX procedure (PROC GLIMMIX, SAS V9.4, SAS Institute, Cary, NC, USA).

## Results

Table 1 shows the general characteristics of the study subjects. Overall, 31,765 (0.55%) patients participated in the pilot program for chronic diseases. There were 18,419 male participants (0.64%) and 13,346 female participants (0.46%). The participation rate was the highest among those aged 40–49 years (0.82%). Patients with higher CCI scores were more likely to participate in the pilot program. When the study population was divided by gender, a similar pattern was shown. The participation rate was high among both males and females aged 40–49 years.

**Table 1:** Characteristics of the study subjects

Variables	Total		Men		Women	
	Total^a^	Participants^b^	Total	Participants	Total	Participants
Total	5,773,687	31,765 (0.55)	2,896,308	18,419 (0.64)	2,877,379	13,346 (0.46)
Age (years)						
≤ 30	13,064	93 (0.71)	9133	65 (0.71)	3931	28 (0.71)
30–39	76,660	520 (0.68)	58,832	398 (0.68)	17,828	122 (0.68)
40–49	430,906	3530 (0.82)	311,354	2539 (0.82)	119,552	991 (0.83)
50–59	1,259,869	8501 (0.67)	750,889	5206 (0.69)	508,980	3295 (0.65)
60–69	1,759,402	9907 (0.56)	918,544	5726 (0.62)	840,858	4181 (0.50)
70–79	1,344,292	6804 (0.51)	566,579	3428 (0.61)	777,713	3376 (0.43)
80–89	756,931	2235 (0.30)	252,990	1016 (0.40)	503,941	1219 (0.24)
≥ 90	132,563	175 (0.13)	27,987	41 (0.15)	104,576	134 (0.13)
CCI						
0	2,530,844	10,088 (0.40)	1,258,343	5650 (0.45)	1,272,501	4438 (0.35)
1	1,666,283	9849 (0.59)	840,740	5703 (0.68)	825,543	4146 (0.50)
2	878,687	6014 (0.68)	451,526	3627 (0.80)	427,161	2387 (0.56)
3+	697,873	5814 (0.83)	345,699	3439 (0.99)	352,174	2375 (0.67)

^a^Total study subjects in this study, ^b^Participants of the national pilot program for chronic diseases

**Fig. 2:**
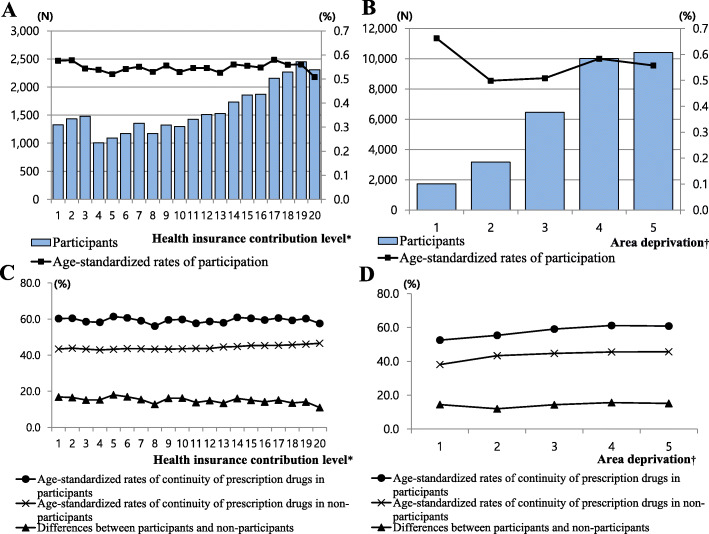
Age-standardized rates of participation and continuity of prescription medication by health insurance contribution level and area deprivation, Note: * The lowest (Q1) and the highest (Q20); † The most-deprived (Q1) and the least-deprived (Q5). **A**: The distribution of age-standardized participation rates by health insurance contribution level. **B**: The distribution of age-standardized participation rates by area deprivation index. **C**: Trend in age-standardized prescription medication continuity by health insurance contribution level and participation status **D**: Trend in age-standardized prescription medication continuity by area deprivation index and participation status

Figure 2 presents the age-standardized rates of participation and prescription medication continuity according to socioeconomic position. There was no clear difference in the age-standardized participation rates based on health insurance contribution level or area deprivation. The participation rates in the lowest and highest insurance contribution vigintile were 0.58 and 0.51%, respectively. The participation rate of patients in the most deprived area and the least deprived were 0.66 and 0.56%, respectively. The number of participants tended to be lower in the more deprived areas and among those with lower health insurance contribution levels (Fig. 2A, B). Regarding urbanization level, 18,900 (0.72%) participants resided in a metropolis, followed by participants from rural areas 2536 (0.51%), and participants from small-to-medium sized cities 10,329 (0.39%) (Supplemental Table 1).

The age-standardized continuity of prescription medication rate for all study subjects was 44.72%; 59.53% for participants and 44.64% for non-participants (Supplemental Table 2). The rate improved approximately 15% according to participation status. Among participants, the rate did not show any trend based on insurance contribution level. However, among non-participants, the higher the insurance contribution level, the higher the rate of prescription medication continuity (Fig. 2C). According to area deprivation, the rate of prescription medication continuity among people living in less deprived areas tended to be higher compared with those living in severely deprived areas. The rates in the least and most deprived areas were 60.83 and 52.54%, respectively. The difference between the two groups was 8.3% points.

Table 2 shows the results of a multilevel logistic regression for a non-participation group and participation group, respectively. In the non-participation group, we found an association between prescription medication continuity and socioeconomic position. Continuity of prescription medication was significantly associated with insurance contribution level. This implied that patients with higher income were likely to continue with prescriptions. Also, the less deprived the area, the higher likelihood of continuity of prescription medication. In Model 2, women were more likely than men to continue with their prescriptions (OR: 1.09, 95% CI: 1.08–1.09). Older individuals had a higher likelihood of continuity of prescription medication. Compared with patients with a Charlson score of 0, those with a Charlson score of 1 or higher showed greater continuity of prescription medication.

**Table 2:** Association between continuity of prescription medication and socioeconomic characteristics in non-participants and participants (Odds Ratio (95% Confidence Interval))

	Non-participants		Participants	
Variables	Model 1	Model 2	Model 1	Model 2
Individual level				
Gender				
Men	1	1	1	1
Women	1.09 (1.08–1.09)	1.09 (1.08–1.09)	1.11 (1.06–1.16)	1.11 (1.06–1.16)
Age	1.01 (1.01–1.02)	1.01 (1.01–1.02)	1.02 (1.02–1.03)	1.02 (1.02–1.03)
CCI				
0	1	1	1	1
1	1.08 (1.08–1.09)	1.08 (1.08–1.09)	0.94 (0.88–1.02)	0.95 (0.88–1.02)
2	1.10 (1.10–1.11)	1.10 (1.10–1.11)	0.99 (0.93–1.07)	0.99 (0.93–1.07)
3+	1.08 (1.08–1.09)	1.08 (1.08–1.09)	0.99 (0.93–1.05)	0.99 (0.93–1.05)
Health insurance contribution level	1.04 (1.04–1.05)	1.04 (1.04–1.05)	0.99 (0.99–1.00)	0.99 (0.99–1.00)
Regional level				
Area deprivation		1.07 (1.07–1.08)		1.08 (1.03–1.12)

Model 1: gender, age, CCI, and health insurance contribution level, Model 2: Model 1 + the area deprivation index

The association between continuity of prescription medication and socioeconomic position of the participation group is also shown in Table 2. There was no statistically significant likelihood of continuing prescriptions based on health insurance contribution. However, as the degree of deprivation decreased, prescription medication continuity increased. In addition, continuity of prescription medications differed according to gender and age.

## Discussion

We studied the effect of prescription medication continuity according to the pilot program for chronic diseases considering socioeconomic position. We found no inequality in participation, and that in the participation group, there was no inequality in continuity of prescription medication according to individual-level socioeconomic position. In both the participation and non-participation group, there were inequalities in prescription medication continuity in terms of area-level socioeconomic position.

The age-standardized participation rate was 0.55% and showed no trend based on health insurance contribution level or area deprivation. While encouraging that the participation rate did not differ according to socioeconomic position, a low participation rate makes it difficult to conclude that there was no inequality in participation rates.

The age-standardized prescription medication continuity rates increased by approximately 15% when people participated in the pilot program for chronic diseases. This suggests that the pilot program was effective. The main purpose of this program was to focus on managing diabetes and hypertension through continuous monitoring. Patients’ blood pressure and blood sugar levels were checked more than once a week, and the results were sent to the clinics. A healthcare professional provided feedback via text messages. If necessary, a telephone consultation including recommended medication or lifestyle changes would be conducted [29]. These services may encourage the continued use of prescriptions. Several studies found that programs assisting patients with uncontrolled hypertension (i.e., blood pressure levels ≥140/90 mmHg) helped lower the level of blood pressure control. These programs taught patients how to measure blood pressure at home, and helped them improve their medication adherence and lifestyle [45–47]. Also, patients who received regular blood pressure telemonitoring interventions had better adherence to anti-hypertensive medication than patients receiving standard care [48].

According to a qualitative analysis of the pilot program for chronic diseases in Korea [30], participants were more likely recognize themselves as healthcare subjects and took responsibility for their health. They realized the importance of managing their lifestyle at home and work while monitoring changes in blood pressure and blood glucose level. These changes could also have positive effects on the health of participants. A previous study identified that education programs solely focused on drug adherence, physical exercise, or smoking cessation for self-management did not improve long-term glycemic control [49]. Patients with hypertension and diabetic complications who participated in the Patient Empowerment Programme (PEP) showed better clinical metabolic control outcomes than non-PEP patients [50]. Patient empowerment can be defined as the process of becoming autonomous through enhancing one’s ability to think critically and make informed decisions when altering behavior [51]. Patients should be internally motivated to manage their health, not dependent on external factors [52]. Thus, it would be preferable if the pilot program for chronic diseases provided services to educate patients on hypertension and diabetes, review test results, and develop skills in program-solving, goal setting, coping, and stress management [53].

Regarding socioeconomic position at the individual level, our findings showed that the pilot program did not worsen health inequality within the participation group. Non-participants with higher health insurance contribution levels had greater continuity with prescriptions compared to those with lower levels. However, there is no significant association between continuity of prescription medication and health insurance contribution level in the participation group. This pilot program was exempted from patient copayments. In 2018, participants received an average of 24 USD per month [54], a financial incentive designed to help people who need to visit clinics regularly. A study examining the financial burden of healthcare services for low-income individuals in Korea reported that people in the lowest-income quintile spent six times more on out-of-pocket payments than those in the highest-income quintile, because the lowest level had a higher prevalence of chronic disease [55]. People with high-income used more prescription medications and spent more money on prescriptions, while reporting less financial burden from prescription medications [56] than people with a low income. A previous study found that medication adherence appeared to decrease with increasing patient copayments among individuals in low-income areas [57]. Out-of-pocket payments could interfere with access to prescription medication and lead to the discontinuation of medication [58]. Therefore, low-income patients may be more reliant on financial aid.

In addition, patients who participated in the pilot program could receive phone consultations to support lifestyle improvements, and doctors evaluated their health status through monitoring. One advantage of this pilot program was providing consultations on risk factors. There is a knowledge gap between people of different socioeconomic positions based on information flow [59]. People from low-socioeconomic groups had less exposure to information on preventing chronic diseases [60] and less awareness of the causes of diseases, which could lead to health disparities [61]. In the light of previous research, the information provided in the pilot program could help manage chronic diseases among patients of a low-socioeconomic status.

Patients living in less deprived areas were more likely to have continuity of prescription medication in both the participation and non-participation groups, which implies that there could be health inequalities based on area deprivation regardless of participation status. A previous study identified that medication adherence was lower among patients in low-income areas compared to those in high-income areas [57]. Health disparities between areas were identified using compositional and contextual effects [62]. Our finding showed that there were still discrepancies in prescription medication continuity between areas after adjusting for individual characteristics. Several studies have reported that the socioeconomic position of a region influenced health disparities between individuals [63–67]. Social and physical features in one neighborhood may affect individual health or access to opportunities for healthy living [62].

Unequal distribution of socioeconomic resources at the regional level is one of the important mechanisms leading social inequalities. There are fewer resources such as good schools or parks in disadvantaged neighborhoods [68]. Residents in poorer areas may have fewer job opportunities as well [69]. Similarly, inequalities in the distribution of healthcare resources may lead to differences in medication adherence. A study investigating the distribution of healthcare resources by classifying regional economic levels based on local tax per person in Korea reported that healthcare resources including the number of specialists, pharmacists, clinics, and pharmacies were advantageously distributed in richer areas [70]. Patients in poorer areas tended to receive less primary healthcare than patients in richer areas [71]. In addition to a quantitative shortage of resources, there may be a lack of quality resources (i.e., health facilities, personnel, and equipment) to meet the needs of people in deprived areas [72]. Further research on the association between the distribution of regional healthcare resources and medication adherence based on socioeconomic position in Korea is needed.

Our study has some limitations. First, we could not consider the educational level and health behaviors related to chronic diseases [73–76] because the National Health Insurance data does not include this information. Second, patients who participated in the pilot program for chronic diseases may be more motivated to manage their health [50]. The degree of effect may have been greater in the pilot program. However, it is difficult to conclude that such an effect made a difference according to the socioeconomic position. Third, distribution of regional healthcare resources was not considered. However, clinics were included as random effects to adjust for variation. Despite these limitations, our study used National Health Insurance data that covered most of the Korean population. To our knowledge, this study was the first to identify whether the pilot program for chronic diseases contributed to reducing health inequalities based on the socioeconomic position at the individual and regional levels.

## Conclusions

There was no difference in the program participation rate at the individual or regional socioeconomic levels in this pilot program. Our findings suggested that the pilot program at the least did not worsen health inequalities at the individual-level socioeconomic position. There was, however, a trend showing health inequalities based on the area-level socioeconomic position. The actual effect of policy intervention may vary depending on socioeconomic position. Therefore, it is necessary to consider the equity perspective in the management of chronic diseases.

## Supplementary Information

**Additional file 1: Supplemental Table 1.** The distribution of age-standardized participation rates by socioeconomic characteristics. Note: Area Deprivation: the most-deprived (Q1); the least-deprived (Q5). **Supplemental Table 2.** The distribution of age-standardized continuity of prescription medication rates by socioeconomic characteristics and participation status. Note: Area Deprivation: the most-deprived (Q1); the least-deprived (Q5).

## Data Availability

Data is available upon request from the Korean National Health Insurance Service (NHIS). (https://nhiss.nhis.or.kr/bd/ab/bdaba000eng.do).

## Abbreviations

CCI: Charlson comorbidity index
HI: Horizontal inequity index
ICD-10: International Classification of Diseases version 10
NHID: National Health Information Database
NHI: National Health Insurance
NHIS: National Health Insurance Service
PEP: Patient empowerment programme
