# Supplementary material for: A national pilot program for chronic diseases and health inequalities in South Korea
Source: BMC Public Health. 2021 Jun 15;21:1142. doi: 10.1186/s12889-021-11208-7 (PMC8204519; doi:10.1186/s12889-021-11208-7)
Supplement: Supplementary file 1 — Additional file 1: Supplemental Table 1. The distribution of age-standardized participation rates by socioeconomic characteristics. Note: Area Deprivation: the most-deprived (Q1); the least-deprived (Q5). Supplemental Table 2. The distribution of age-standardized continuity of prescription medication rates by socioeconomic characteristics and participation status. Note: Area Deprivation: the most-deprived (Q1); the least-deprived (Q5). [file 12889_2021_11208_MOESM1_ESM.docx]

**Supplemental Table 1** The distribution of age-standardized participation rates by socioeconomic characteristics

| Variables | Total study subjects | Participants | Standardized rate (%) |
| --- | --- | --- | --- |
| Total | 5,773,687 | 31,765 | (0.55) |
| Health insurance contribution level | |  |  |
| 1 | 250,584 | 1,327 | (0.58) |
| 2 | 259,218 | 1,435 | (0.58) |
| 3 | 256,829 | 1,479 | (0.54) |
| 4 | 179,914 | 1,006 | (0.54) |
| 5 | 200,264 | 1,091 | (0.52) |
| 6 | 206,610 | 1,170 | (0.54) |
| 7 | 233,350 | 1,356 | (0.55) |
| 8 | 212,276 | 1,172 | (0.53) |
| 9 | 229,741 | 1,325 | (0.56) |
| 10 | 234,323 | 1,294 | (0.53) |
| 11 | 251,994 | 1,425 | (0.55) |
| 12 | 266,439 | 1,511 | (0.55) |
| 13 | 282,576 | 1,529 | (0.53) |
| 14 | 303,000 | 1,732 | (0.56) |
| 15 | 330,444 | 1,859 | (0.55) |
| 16 | 337,718 | 1,873 | (0.55) |
| 17 | 378,108 | 2,158 | (0.58) |
| 18 | 421,651 | 2,265 | (0.56) |
| 19 | 461,411 | 2,450 | (0.56) |
| 20 | 477,237 | 2,308 | (0.51) |
| Difference |  |  | -(0.07) |
| Urbanization level |  |  |  |
| Metropolitan | 2,595,342 | 18,900 | (0.72) |
| Urban | 2,631,209 | 10,329 | (0.39) |
| Rural | 547,136 | 2,536 | (0.51) |
| Difference |  |  | (0.21) |
| Area Deprivation |  |  |  |
| 1st quintile | 313,171 | 1,729 | (0.66) |
| 2nd quintile | 674,577 | 3,165 | (0.50) |
| 3rd quintile | 1,276,863 | 6,451 | (0.51) |
| 4th quintile | 1,699,025 | 10,012 | (0.58) |
| 5th quintile | 1,810,051 | 10,408 | (0.56) |
| Difference |  |  | -(0.10) |

**Note:** Area Deprivation: the most-deprived (Q1); the least-deprived (Q5)

**Supplemental Table 2** The distribution of age-standardized continuity of prescription medication rates by socioeconomic characteristics and participation status

| Variables | | Total | | |  | Participation | | |  | Non-participation | | |
| --- | --- | --- | --- | --- | --- | --- | --- | --- | --- | --- | --- | --- |
|  |  | Patients with continuity of prescription medication | | Standardized rate |  | Patients with continuity of prescription medication | | Standardized rate |  | Patients with continuity of prescription medication | Standardized  rate | |
| Total | | 2,582,064 | | (44.72) |  | 18,910 | | (59.53) |  | 2,563,154 | (44.64) | |
| Health insurance contribution level | | | | | | | | | | | | |
| 1 | | | 111,448 | (43.54) |  | 814 | (60.30) | |  | 110,634 | | (43.44) |
| 2 | | | 116,127 | (43.99) |  | 875 | (60.48) | |  | 115,252 | | (43.89) |
| 3 | | | 110,755 | (43.47) |  | 859 | (58.60) | |  | 109,896 | | (43.39) |
| 4 | | | 76,642 | (43.02) |  | 582 | (58.22) | |  | 76,060 | | (42.94) |
| 5 | | | 86,927 | (43.43) |  | 668 | (61.37) | |  | 86,259 | | (43.34) |
| 6 | | | 90,836 | (43.82) |  | 713 | (60.69) | |  | 90,123 | | (43.73) |
| 7 | | | 101,409 | (43.65) |  | 797 | (59.10) | |  | 100,612 | | (43.60) |
| 8 | | | 91,577 | (43.42) |  | 661 | (56.11) | |  | 90,916 | | (43.35) |
| 9 | | | 98,636 | (43.44) |  | 781 | (59.56) | |  | 97,855 | | (43.35) |
| 10 | | | 101,406 | (43.65) |  | 765 | (59.86) | |  | 100,641 | | (43.57) |
| 11 | | | 109,753 | (43.87) |  | 815 | (57.64) | |  | 108,938 | | (43.80) |
| 12 | | | 116,130 | (43.88) |  | 877 | (58.71) | |  | 115,253 | | (43.80) |
| 13 | | | 125,633 | (44.64) |  | 878 | (57.86) | |  | 124,755 | | (44.57) |
| 14 | | | 135,961 | (44.95) |  | 1,055 | (60.91) | |  | 134,906 | | (44.86) |
| 15 | | | 149,988 | (45.44) |  | 1,116 | (60.43) | |  | 148,872 | | (45.35) |
| 16 | | | 153,687 | (45.40) |  | 1,119 | (59.49) | |  | 152,568 | | (45.33) |
| 17 | | | 172,996 | (45.56) |  | 1,308 | (60.63) | |  | 171,688 | | (45.48) |
| 18 | | | 193,672 | (45.79) |  | 1,360 | (59.30) | |  | 192,312 | | (45.71) |
| 19 | | | 214,100 | (46.24) |  | 1,494 | (60.32) | |  | 212,606 | | (46.16) |
| 20 | | | 224,381 | (46.64) |  | 1,373 | (57.59) | |  | 223,008 | | (46.58) |
| Difference | | |  | (3.10) |  |  | -(2.71) | |  |  | | (3.14) |
| Urbanization level | | | |  |  |  |  | |  |  | |  |
| Metropolitan | 1,193,933 | | | (45.93) |  | 11,414 | (60.21) | |  | 1,182,519 | | (45.82) |
| Urban | 1,168,344 | | | (44.62) |  | 6,135 | (60.06) | |  | 1,162,209 | | (44.56) |
| Rural | 219,787 | | | (40.06) |  | 1,361 | (53.22) | |  | 218,426 | | (40.00) |
| Difference |  | | | (5.86) |  |  | (6.99) | |  |  | | (5.83) |
| Area Deprivation |  | | |  |  |  |  | |  |  | |  |
| 1st quintile | 119,973 | | | (38.20) |  | 916 | (52.54) | |  | 119,057 | | (38.10) |
| 2nd quintile | 296,243 | | | (43.42) |  | 1,775 | (55.31) | |  | 294,468 | | (43.36) |
| 3rd quintile | 573,530 | | | (44.76) |  | 3,814 | (59.02) | |  | 569,716 | | (44.68) |
| 4th quintile | 774,261 | | | (45.60) |  | 6,124 | (61.15) | |  | 768,137 | | (45.51) |
| 5th quintile | 818,057 | | | (45.77) |  | 6,281 | (60.83) | |  | 811,776 | | (45.68) |
| Difference |  | | | (7.57) |  |  | (8.29) | |  |  | | (7.58) |

**Note:** Area Deprivation: the most-deprived (Q1); the least-deprived (Q5)
